# Chip-In-Loop SNN Proxy Learning: a new method for efficient training of spiking neural networks

**DOI:** 10.3389/fnins.2023.1323121

**Published:** 2024-01-04

**Authors:** Yuhang Liu, Tingyu Liu, Yalun Hu, Wei Liao, Yannan Xing, Sadique Sheik, Ning Qiao

**Affiliations:** ^1^SynSense Co. Ltd., Chengdu, China; ^2^SynSense AG., Zurich, Switzerland

**Keywords:** SNN, asynchronous, neuromorphic chip, CIL-SPL, event-driven

## Abstract

The primary approaches used to train spiking neural networks (SNNs) involve either training artificial neural networks (ANNs) first and then transforming them into SNNs, or directly training SNNs using surrogate gradient techniques. Nevertheless, both of these methods encounter a shared challenge: they rely on frame-based methodologies, where asynchronous events are gathered into synchronous frames for computation. This strays from the authentic asynchronous, event-driven nature of SNNs, resulting in notable performance degradation when deploying the trained models on SNN simulators or hardware chips for real-time asynchronous computation. To eliminate this performance degradation, we propose a hardware-based SNN proxy learning method that is called Chip-In-Loop SNN Proxy Learning (CIL-SPL). This approach effectively eliminates the performance degradation caused by the mismatch between synchronous and asynchronous computations. To demonstrate the effectiveness of our method, we trained models using public datasets such as N-MNIST and tested them on the SNN simulator or hardware chip, comparing our results to those classical training methods.

## 1 Introduction

Spiking neural networks (SNNs) is a new generation neural network based approach for neuromorphic computing owing to their low power consumption and high efficiency (Merolla et al., 2014). SNNs, inspired by biological neurons, use discrete spikes to transmit information, allowing them to process asynchronous, event-driven data efficiently (Maass, 1997; Ponulak and Kasinski, 2011). SNNs are also good at handling spatio-temporal information, offering improved performance for dynamic tasks and time-sensitive problems (Pfeiffer and Pfeil, 2018; Zhang et al., 2018).

Nevertheless, despite these advantages, SNNs have not gained widespread adoption primarily due to the lack of efficient training methods, which contrasts with the relatively straightforward training of traditional artificial neural networks (ANNs) (Neftci et al., 2019). This challenge arises from the discontinuous and non-differentiable nature of spike signals, which complicates the application of popular gradient-based optimization techniques such as back propagation (Rumelhart et al., 1986; Bengio et al., 2015). Alternative training methods, such as surrogate gradients or spike-timing-dependent plasticity (STDP) (Zhang et al., 2021), frequently result in slower convergence and reduced accuracy when compared to ANNs (Diehl and Cook, 2015; Neftci et al., 2019).

The most common methods for training SNNs involve either training ANNs and then converting them to SNNs (Diehl and Cook, 2015) or directly training SNNs using surrogate gradient methods (Neftci et al., 2019). Both of these methods suffer from a common problem: they rely on frame-based approaches that accumulate asynchronous events into synchronous frames and perform computations within frames. This deviation from the true asynchronous, event-driven nature of SNNs leads to significant performance degradation when the trained models are deployed on SNN simulators or hardware chips (Benjamin et al., 2014; Davies et al., 2018) for real-time asynchronous computation.

The necessity to adapt to asynchronous computation stems from the fundamental differences between SNNs and ANNs. Unlike ANNs, SNNs operate on an asynchronous basis, where computations are triggered by individual neural events rather than predetermined frames. This asynchronous processing is key to the low-power consumption and reduced latency that characterizes SNNs. The performance degradation, which is caused by the differences of synchronous computation and asynchronous computation, is difficult to effectively compensate for or eliminate it, which poses a major obstacle for the practical application of SNNs.

In this study, we propose a hardware-based SNN proxy learning method to minimize the mismatch for SNNs running on sync/async platforms. The primary concept behind this approach involves using the time-step based backward propagation graph as a substitute for asynchronous inference outputs. Gradients are consequently computed from asynchronous system outputs, presuming a linear correlation between ReLU activations and spiking neuron firing rates.

The remainder of this study is organized as follows, Section 2 describes an overview of the existing methods for SNN training and their limitations as well as a brief review of the recent advancements in the field. Section 3 illustrates the proposed Chip-In-Loop SNN Proxy Learning (CIL-SPL) method, including its main components, algorithms, and implementation details. Section 4 introduces the experiments conducted using public datasets such as N-MNIST and the performance comparison between the proposed method and the classical training methods on SNN simulator or the hardware chip. Finally, Section 5 discusses the implications of our findings, the strengths and weaknesses of the proposed method, and potential future directions for research in this area.

## 2 Related studies

In this section, we will mainly introduce related studies about SNN training methods as follows.

### 2.1 Conversion method

The method of training an artificial neural network (ANN) and converting it to an SNN has been adopted widely (Diehl and Cook, 2015; Rueckauer et al., 2017; Rathi et al., 2020). The advantage of this method lies in the fact that the training methods for ANNs have been extensively optimized and researched over a long period, offering greater usability. Moreover, the training process is more straightforward, converges faster, and can achieve good results. This conversion, however, has its limitations as the original ANNs do not consider the spatio-temporal dynamics of SNNs, and this can lead to performance degradation (Rueckauer et al., 2017). This degradation is inherently challenging to eliminate due to the fundamental differences between SNNs and ANNs, ensuring that any optimizations applied to the ANN yield limited improvements.

### 2.2 Direct training method

Another alternative is using surrogate gradients (Neftci et al., 2019; Fang et al., 2021). Surrogate gradient methods address the non-differentiability of spike events in SNNs by using approximated gradients. This allows for conventional optimization, improved convergence, and broader applicability in SNN training. but these methods still face the issue of slow convergence and lower accuracy compared to ANNs. Moreover, some direct training methods such as back propagation through time (BPTT) (Lee et al., 2016; Bellec et al., 2018; Neftci et al., 2019) or real-time recurrent learning (RTRL) (Williams and Zipser, 1989; Pedroni et al., 2016), which incorporate time-wise gradient optimization for more efficient gradient computation in SNNs, have been also utilized for SNNs. The advantage of these methods is that they lead less performance degradation during the conversion (Wei et al., 2023). However, these methods are computationally expensive and hard to scale.

### 2.3 Proxy learning and proxy learning in SNNs

Proxy learning has proven effective in training deep neural networks. It involves training an easier-to-optimize proxy model and then transferring the learned weights to the target model (Romero et al., 2014). This strategy has helped overcome problems such as vanishing gradients in deep learning architectures. Some recent studies have applied the concept of proxy learning to SNNs. Kheradpisheh et al. (2022) proposed spike-based proxy training for deep SNNs. They backpropagate the mismatch of the SNN in the proxy ANN to update the shared weights, simply by replacing the ANN final output with that of the SNN. Wu et al. (2021a,b) proposed a learning method that is called tandem learning. This method can be viewed as forms of proxy learning but with an emphasis on collaboration and synchronization during the training process.

### 2.4 Hardware-specific training of SNNs

Considering hardware dynamics during training has also been an area of interest. Methods such as SLAYER (Shrestha and Orchard, 2018; Xing et al., 2020) and Whetstone (Severa et al., 2018) take into account the specific characteristics of neuromorphic hardware during training. However, these methods also use approximations to achieve this, leading to potential inaccuracies.

Inspired by these studies, we propose Chip-In-Loop SNN Proxy Learning (CIL-SPL), a hardware-based SNN proxy learning method that eliminates performance degradation by maintaining the true asynchronous nature of SNNs during training. Additionally, our method can be integrated with various training methods. We demonstrate its effectiveness by training models on public datasets and deploying them on both SNN simulators and hardware chips.

## 3 Chip-In-Loop SNN Proxy Learning

**Figure 1:**
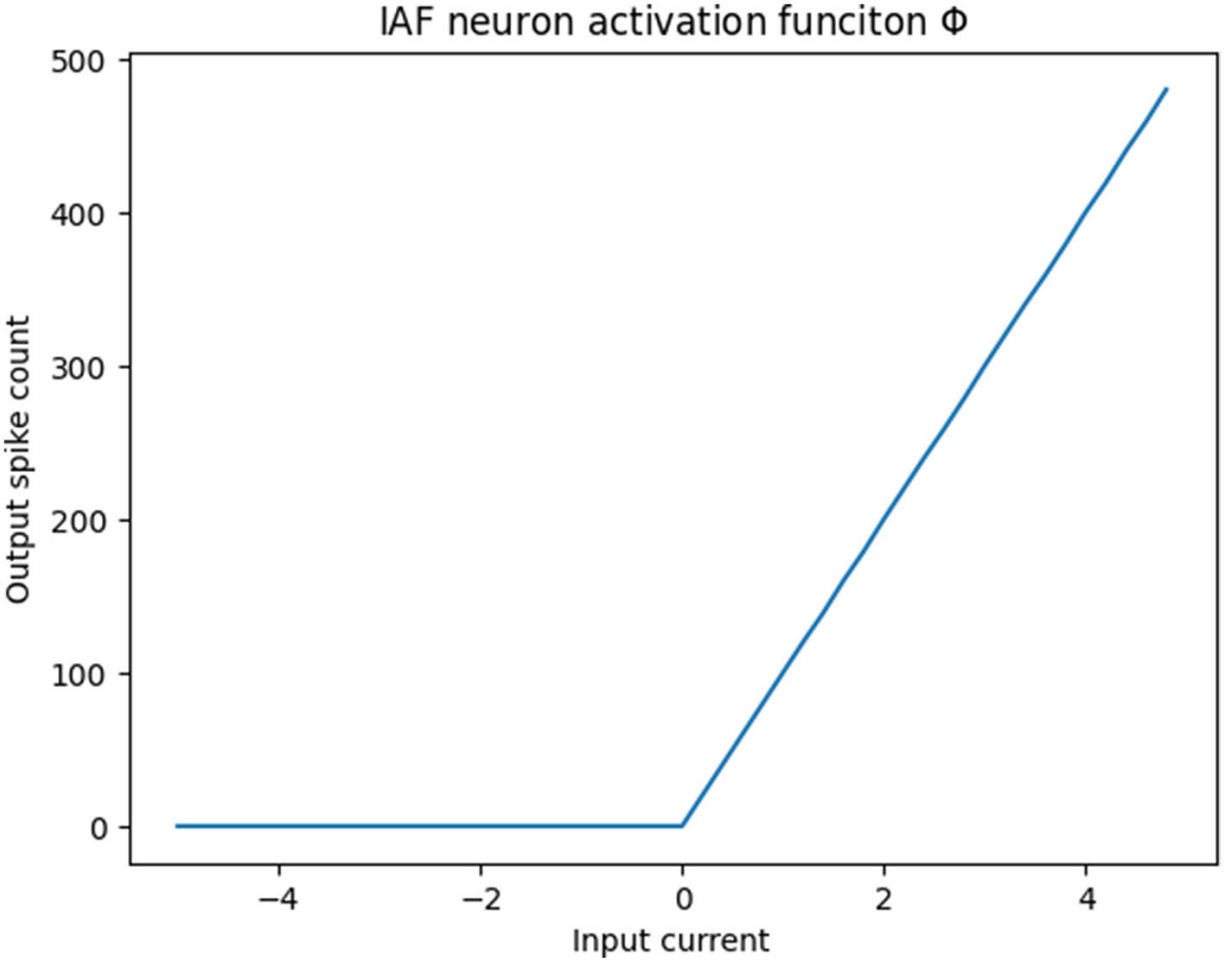
IAF response regards to the synaptic current vs. firing rate.

In this study, we introduce the Chip-In-Loop SNN Proxy Learning (CIL-SPL), a novel SNN training method that emphasizes real-world, event-driven, asynchronous behavior by leveraging hardware integration and possesses the unique flexibility to fuse with various training approaches including ANN-to-SNN conversion and the BPTT method. The CIL-SPL method follows the logic of proxy learning and incorporates the hardware chip (or simulator) as a proxy agent. The input events would be forwarded by the hardware device or its simulator and the loss gradients backward by the same synchronous SNN structure. The computation graph is thus shared in the backward stage using the traditional gradient decent method. This relies on the fact that the ReLU activation is linear corresponding to the spiking neuron firing rate within a limited time window. As is shown in the Figure 1, the response of the integrate-and-fire neuron (IAF) can be likened to a sampled ReLU activation function albeit in a different scale. Leveraging this concept, the spike counts of asynchronous neuron outputs within a timestep can serve as an approximate activation value, essentially acting as a proxy within the standard time-step based computational structure. Regarding the temporal domain, given that the actual computation occurs continuously and asynchronously, the loss is fragmented into small time windows, accounting for accumulated spike count errors. This can be addressed by employing backpropagation through time (BPTT) for resolution.

### 3.1 Hardware introduction: Speck^tm^ chip and its simulator

Speck^TM^ is a “sensor-compute integrated” neuromorphic intelligent dynamic vision System on Chip(SoC), integrates a dynamic vision processor (DYNAP^tm^CNN) and a dynamic vision sensor (DVS) (Delbruck et al., 2008; Gallego et al., 2020; Liao et al., 2022), also known as event camera. It features a large-scale spiking convolutional neural network (SCNN) chip architecture based on an asynchronous logic paradigm, configurable with up to 320K spiking neurons. In the processing core, only the address event representation (AER) protocol is used and all the neuron dynamics are purely asynchronous event-driven without a local/global clock reference signal.

Since our experiments primarily utilize the computational core of Speck^tm^ chip, we will mainly introduce the key features of the core rather than the DVS part. The computational core has nine DYNAP^tm^CNN layers, each layer consists of asynchronous convolution layers, spiking neurons, and pooling layers. It offers a variety of stride, padding, and pooling options to cater to different network structures and application needs. As for the specific network size and the number of parameters, the parameter count for the convolutional kernels and spiking neurons in each layer varies, approximately in the tens of Ks. The precision for the convolutional kernel precision is 8 bits, while the precision for the spiking neuron states is 16 bits.

As for the simulator, it emulates based entirely on the chip's architecture. Due to the electrical variations of the actual chip, there might be minor discrepancies. We conducted experiments using both the hardware chip and the simulator, with consistent experimental results.

By utilizing the speck development board and its accompanying host computer software, data can be flexibly transmitted for computation and reading of intermediate data or results. This enables an equivalent convenient asynchronous neural network simulation on both actual hardware and host machine.

### 3.2 Spiking neuron and network structure

As for the spiking neurons, we conducted experiments using both integrate and fire (IF) neurons and the leaky integrate and fire neurons (Abbott, 1999; Gerstner and Kistler, 2002; Izhikevich, 2003).

The principle of IF neuron could be described by


(1) $$\begin{array}{l} \begin{array}{c} V_{\text{mem}}\left(t+1\right)=V_{\text{mem}}\left(t\right)+\sum z\left(t\right)\\ \text{if}V_{\text{mem}}\left(t\right)\geq V_{\text{th}},\text{then}V_{\text{mem}}\to V_{\text{reset}} \end{array} \end{array}$$

where ∑*z*(*t*) is the sum of input currents and *V*_*mem*_(*t*) is the membrane potential at time *t*. When the membrane potential *V*_mem_(*t*) reaches or exceeds the threshold *V*_th_, the IF neuron will output a spike and then reset the membrane potential to *V*_reset_. Based on IF neuron, the LIF neuron has an extra leaky mechanism for membrane potential, which can be described as


(2) $$\begin{array}{l} V_{\text{mem}}(t+1)=\max\left(\alpha V_{\text{mem}}(t)+(1-\alpha )\sum z(t),V_{\text{min}}\right)\\ \text{ if }V_{\text{mem}}(t)\geq V_{\text{th}},\text{ then }V_{\text{mem}}\to V_{\text{reset}} \end{array}$$

where $\alpha =e^{-\frac{1}{\tau_{\text{mem}}}}$ is the leakage factor dominated by the time constant factor τ_mem_. It means that the membrane potential at time t+1, i.e., *V*_mem_(*t*+1) is a linear combination of the previous membrane potential *V*_mem_(*t*) and the sum of input currents ∑*z*(*t*), weighted by the coefficient α. As time pass by, *V*_mem_(*t*+1) will decay with α as the coefficient. Therefore, the LIF neuron is more complex than IF neuron, by incorporating a leakage mechanism.

As for the network structure, we conducted experiments on an SCNN (Diehl et al., 2015) with six layers, including five spiking convolutional layers and one full-connection layer. The network could be represented as follows:


$$\begin{array}{l} \text{SCNN Structure:}\\ \underset{\text{Spiking Convolutional Layer (1)}}{\underset{︸}{\text{Conv (Async)}\to \text{Activation (Spiking)}\to \text{Pooling}}}\\ \underset{\text{Spiking Convolutional Layer (2)}}{\underset{︸}{\text{Conv (Async)}\to \text{Activation (Spiking)}\to \text{Pooling}}}\\ \vdots \\ \underset{\text{Spiking Convolutional Layer (5)}}{\underset{︸}{\text{Conv (Async)}\to \text{Activation (Spiking)}\to \text{Pooling}}}\\ ↓\\ \text{Fully Connected Layer} \end{array}$$

Notably, this SCNN does not incorporate any biases. Mathematically, for each *i*th spiking convolutional layer, the operation can be described as


$$O_{i}=P\left(S\left(I_{i}*K_{i}\right)\right)$$

where *I*_*i*_ is the input of *i*th layer, *K*_*i*_ is the convolution kernel, *S*(·) is the spiking activation function, and *P*(·) is the pooling operation.

The output from the fully connected layer, followed by the spiking activation, can be represented as


$$F=S\left(W\cdot O_{5}\right)$$

where *W* is the weight matrix and *O*_5_ is the output from the last (5th) spiking convolutional layer.

### 3.3 CIL-SPL structure

**Figure 2:**
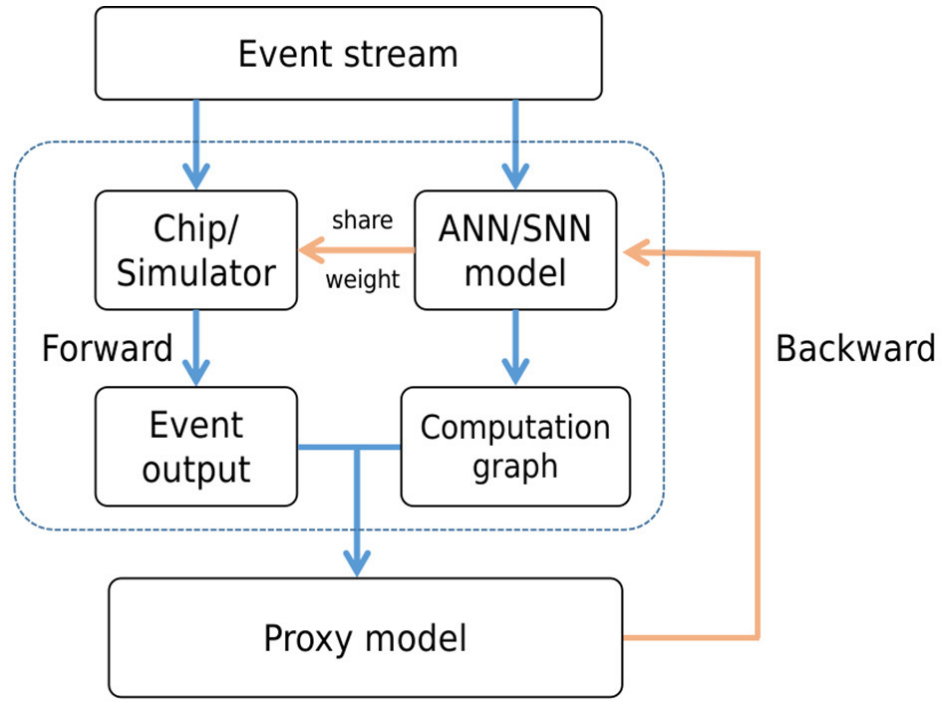
The structure of CIL-SPL. The event stream is computed by both hardware chip and ANN/SNN model, which obtain the event output by chip and computational graph by model. Then, the result from the hardware would be transferred into the computational graph and backward by ANN/SNN model. Finally, the weights would be shared with the on-chip model after back propagation updating.

CIL-SPL follows the base structure of proxy learning, and the model will run on synchronous software framework and also hardware chip (simulator) to be the proxy agent. In each iteration, the input events will be fed into the chip and carry out the asynchronous forward computation on the chip. Meanwhile, the events would also be sent into a synchronous software framework and then be converted into tensors by accumulating events over a period of time for forward computation. For loss computation and gradient backpropagation, we replace the outputs of the standard network with the chip's output. This means the loss would be calculated by the output of the chip and proceed with backpropagation in the original synchronous computation graph. As is shown in Figure 2, the gradient computation still occurs within the synchronous computational graph. However, for the loss calculation, the output of the asynchronous framework is used in place of the synchronous output. The asynchronous framework is responsible only for forward computation and does not independently compute the loss or update the gradients. After the backward process, the updated weights would be transferred to the chip and then start the next iteration.

The fundamental concept involves leveraging the asynchronous computation outcomes from the hardware chip to substitute the synchronous computation results within the original training framework during the forward stage of each iteration. During the backward stage, the gradient backpropagation process persists within the original ANN/SNN computation graph to fine-tune the weights. Subsequently, these refined weights are transmitted back to the hardware chip in preparation for the subsequent iteration. This approach's advantage lies in integrating asynchronous hardware chip computation during the forward stage, which steers the network toward optimizing asynchronous computation results. However, in the backward stage, due to the substantial computational demands of asynchronous gradient computation and the limitation in deriving effective errors from single-step calculations, the original framework remains instrumental for gradient backpropagation, ensuring a balance between efficiency and reliability.

Moreover, given the lower precision of weights on the chip (8 bits or 16 bits), a feasible approach is to quantize the parameters after each iteration when transferring them. A more advantageous method would be to employ quantization-aware training (QAT) within the original training framework, allowing the primary model to adapt to the degradation introduced by quantization during the training process.

In the following, we will illustrate how the CIL-SPL method integrates with different training methods, taking ANN-to-SNN and BPTT as primary examples.

#### 3.3.1 CIL-SPL with ANN-to-SNN

For the ANN-to-SNN training method, the entire training process is conducted on the ANN. Only after the training is completed, the parameters can be transferred to the SNN. For the CIL-SPL with the ANN-to-SNN method, within each proxy loop, both the ANN and SNN receive identical inputs and independently conduct forward computations. Next, the SNN's output substitutes the ANN's output for computing the ANN's loss. Subsequent to this, backward propagation occurs within the ANN to modify the weights. These adjusted weights are then transferred to the SNN, and the process iterates for the next cycle. This algorithmic process can be outlined as Algorithm 1.

**Algorithm 1:** CIL-SPL with ANN-to-SNN.

**Inputs:** *x* (training input data), *y* (training labels)
**Parameters:** $W_{ANN}^{\left(1\right)}$, $W_{SNN}^{\left(1\right)}$ (Initialized weights of the ANN and SNN)
**Hyper-parameters:** α(learning rate), *T*(number of total iterations)
**Functions:** *f*(Forward computation function), *L*(Loss function), Φ(Weights transfer function)
**Start training:**
**for** *t* = 1 to *T* **do**
$y_{ANN}^{\left(t\right)}=f_{ANN}\left(W_{ANN}^{\left(t\right)},x\right)$
$y_{SNN}^{\left(t\right)}=f_{CHIP\text{\_}SNN}\left(W_{SNN}^{\left(t\right)},x\right)$
$\Delta W_{ANN}^{\left(t\right)}=\alpha \nabla_{W_{ANN}^{\left(t\right)}}L\left(y_{SNN}^{\left(t\right)},y\right)$
$W_{ANN}^{\left(t+1\right)}=W_{ANN}^{\left(t\right)}+\Delta W_{ANN}^{\left(t\right)}$
$W_{SNN}^{\left(t+1\right)}=\Phi \left(W_{ANN}^{\left(t+1\right)}\right)$
**end for**
**Output:** Trained weights $W_{ANN}^{\left(T+1\right)}$, $W_{SNN}^{\left(T+1\right)}$

There are several noteworthy points within this process. First, SNN's forward computation occurs on the chip or hardware simulator and is involved in each iteration of the loop. Second, given that the SNN's final output is the accumulated spike count for each category, a softmax function is applied to align the output format with that of the ANN in terms of probability. This alignment facilitates the computation of the loss function. As for the weight conversion from ANN to SNN, due to the lower weight precision on the chip (or simulator), quantization is required. Hence, the transfer method is a quantization function. If the QAT method was already adopted during the training process of the ANN, then the quantized weights can be directly used as the weights for the SNN.

#### 3.3.2 CIL-SPL with BPTT

For the BPTT training method, during each iteration, several time steps are divided. In the forward process, the state of each time step is recorded. During the model optimization process, the states from all previous time steps are used to optimize the weights. In the CIL-SPL with BPTT method, we only need to include the chip (or simulator) during the forward stage to perform simultaneous forward computations. During the loss computation and backpropagation stages, the final output *y*_*chip*_[*t*] and membrane potential *V*_*chip*_[*t*] from the chip are used to replace those in the original synchronous network. Every iteration process can be represented by the following formulas:

- **Forward propagation on synchronous framework:**

(3) $$\begin{array}{l} V\left[t\right]=f_{\text{state}}\left(V\left[t-1\right],x\left[t\right],W\right) \end{array}$$

(4) $$\begin{array}{l} y\left[t\right]=f_{\text{out}}\left(V\left[t\right]\right) \end{array}$$

- **Forward propagation on asynchronous chip:**

(5) $$\begin{array}{l} V_{\text{chip}}\left[t\right]=f_{\text{chip\_state}}\left(V_{\text{chip}}\left[t-1\right],x\left[t\right],W_{\text{chip}}\right) \end{array}$$

(6) $$\begin{array}{l} y_{\text{chip}}\left[t\right]=f_{\text{chip\_out}}\left(V_{\text{chip}}\left[t\right]\right) \end{array}$$

- **Loss calculation:**

(7) $$\begin{array}{l} L\left[t\right]=\overset{t}{\underset{\tau =1}{\sum }}L\left(y_{\text{chip}}\left[\tau \right],y_{\text{true}}\left[\tau \right]\right) \end{array}$$

- **Back propagation:**

(8) $$\begin{array}{l} \frac{\partial L}{\partial V_{\text{chip}}\left[\tau \right]}=\overset{T}{\underset{t=\tau }{\sum }}\frac{\partial L\left[t\right]}{\partial y_{\text{chip}}\left[t\right]}\cdot \frac{\partial y_{\text{chip}}\left[t\right]}{\partial V_{\text{chip}}\left[\tau \right]} \end{array}$$

(9) $$\begin{array}{l} \frac{\partial L}{\partial W}=\overset{T}{\underset{t=1}{\sum }}\frac{\partial L\left[t\right]}{\partial y_{\text{chip}}\left[t\right]}\cdot \frac{\partial y_{\text{chip}}\left[t\right]}{\partial V_{\text{chip}}\left[t\right]}\cdot \frac{\partial V_{\text{chip}}\left[t\right]}{\partial W} \end{array}$$

(10) $$\begin{array}{l} W^{\left(t+1\right)}=W^{\left(t\right)}-\alpha \cdot \frac{\partial L}{\partial W} \end{array}$$

where *x* represents the input, *V* represents the membrane potential, *y* denotes to the output, *W* represents the weights, *L* is the loss function, α is the learning rate, and *f* denotes the forward computation function. Terms with the subscript “chip” refer to corresponding entities in the chip or simulator. Thus, compared to the traditional BPTT method, this approach requires additional forward computations on the chip. During the loss calculation and backpropagation, the membrane potential *V*_chip_ and output *y*_chip_ obtained from the forward pass on the chip are used in place of the original *V* and *y*. As for the parameter initialization and the transfer of parameters after each iteration, it is similar to that in CIL-SPL with ANN-to-SNN and will not be elaborated here again.

This approach is adopted because the typical BPTT method divides the computations into several time steps. However, due to computational resource constraints and gradient optimization efficacy, we can not divide it into too many time steps. Each time step accumulates events over a certain duration or quantity for synchronous computation, which differs from the actual asynchronous computation process on the chip. Therefore, during the backward stage, we replace with the chip's output results and adjust the weights to optimize their influence on the chip's output.

For the CIL-SPL with BPTT method, the chip not only participates in the iterative loop but also joins in the time step loop within each iteration, achieving a true sense of “chip-in-loop.”

Apart from the ANN-to-SNN and BPTT methods, the CIL-SPL approach can also be combined with other training techniques, and even potentially with future novel methods. The core principle is to replace the forward output results in the training method with outputs from the hardware chip (or simulator) during the iteration process, subsequently optimizing the parameters. This ensures that the network optimization is oriented toward the actual asynchronous chip output process and results.

## 4 Experiments and results

### 4.1 Experiments set-up

We conducted experiments on the neuromorphic-MNIST (N-MNIST) dataset (Orchard et al., 2015). The N-MNIST dataset is essentially a spiking version of the conventional MNIST (LeCun et al., 1998), where images are converted into spiking sequences. It consists of the same 60,000 training and 10,000 testing samples as the original MNIST dataset and is captured at the same visual scale as the original MNIST dataset (28 × 28 pixels). To demonstrate the effectiveness of our method, we conducted comparative experiments with our approach against both the CNN and BPTT methods. Moreover, tests were performed in both synchronous software frameworks and asynchronous hardware environments.

First, we conducted experiments using CNN to establish a solid benchmark. During the experimentation, we converted the DVS event stream from the N-MNIST dataset back into image frames for training and testing the CNN. Subsequently, to investigate the effects of quantization during training and testing, we conducted similar experiments using a CNN with quantization-aware training (QAT), setting the weight parameter resolution to 8 bits. Following the training within a software framework using these CNN-based approaches, we employed the ANN-to-SNN conversion method to transition them into SNNs. These transformed networks were then deployed onto hardware chips for testing purposes. Furthermore, we extended the deployment to a synchronous hardware accelerator, facilitating comparative experiments.

For direct SNN training, we conducted experiments using the BPTT method as well as our CIL-SPL with BPTT approach. Initially, we trained and tested on a software framework, and subsequently, we deployed and tested on the hardware chip.

### 4.2 Experimental results and analysis

The experimental results are listed in Table 1. First, it is evident that on the software-side with synchronous computational architecture, both CNN and BPTT methods achieved commendable results. However, when deployed on asynchronous hardware chips, the CNN-to-SNN method suffered a more substantial performance degradation. This is because the multi-time step approach of BPTT is more aligned with the real asynchronous process.

**Table 1:** Software and hardware results of four different methods on N-MNIST dataset (Accuracy: %).

**Method**	**Software test**	**Hardware ANN test**	**Hardware SNN test**
CNN only	97.39	96.89	91.75
CNN with QAT	95.98	93.33	92.15
BPTT(SNN)	97.56	/	95.24
CIL-SPL with BPTT	**96.66**	/	**95.71**

Furthermore, regarding computational precision discrepancies, it is observed that after incorporating the QAT method, while the accuracy of the CNN on the software side slightly decreased, its performance on the hardware SNN chip was significantly enhanced.

As for our CIL-SPL method, it can be observed that, although it did not achieve the best results during training and testing on the software side, its accuracy degradation was the smallest when deployed to the hardware chip, and it achieved the best performance on the chip. This conforms with our expectations. Our proposed training method was not aimed at achieving higher accuracy on general software platforms but rather at reducing performance degradation when deployed on hardware chips due to differences between synchronous and asynchronous computations as well as variations in weight precision.

As for deploying the non-quantized CNN model to the hardware ANN inference accelerator, it achieved higher accuracy compared to when deployed on the hardware SNN chip. However, its power consumption, computational requirements, and required storage space were significantly higher than that of the SNN chip.

### 4.3 Distribution of weights

**Figure 3:**
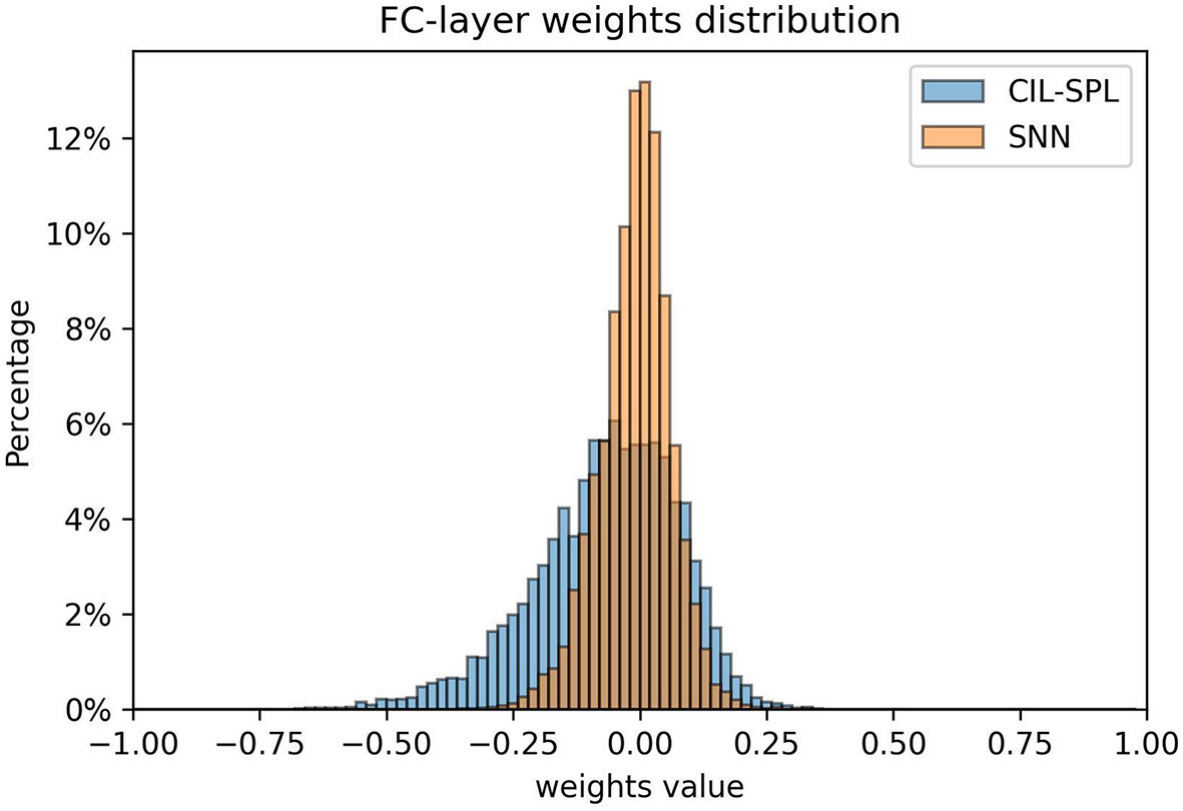
Distribution of fully-connected layer weights comparing the CIL-SPL and SNN structures, the distribution of CIL-SPL is sparse which benefits the quantization after deploying on the chip.

Additionally, we analyzed the model weights trained separately using the BPTT method and the CIL-SPL method. Figure 3 displays the weight distribution of these models.

It could be observed that the model trained using the BPTT method has a denser weight distribution, while the model trained with the CIL-SPL method exhibits a more uniform weight distribution. A denser distribution of weight values necessitates higher precision to distinguish between them. During quantization, if the precision is not sufficient, weights that are close in value may be rounded to the same quantized number, leading to a greater loss of information and potentially impairing the network's performance (Rastegari et al., 2016). This might offer an intuitive explanation as to why models trained using the CIL-SPL method experience smaller degradation when deployed to hardware chips.

## 5 Discussion

In this study, we propose a new method of SNN training called CIL-SPL which aims to reduce the quantization precision loss from high-resolution software to low-resolution hardware and benchmark it on the N-MNIST data set.

In our exploration of the Chip-In-Loop SNN Proxy Learning (CIL-SPL) approach, we primarily sought to reconcile the discrepancies between synchronous training and true asynchronous inference on hardware. Notably, CIL-SPL is versatile, seamlessly integrating with various training strategies, for example, the ANN-to-SNN conversion method and the BPTT direct training method. Our method demonstrated remarkable results, achieving 95.71% accuracy on the N-MNIST dataset, which is conducted on SNN hardware chip. This impressive experimental result was attained with minimized network parameters and parameter precision on hardware, substantiating CIL-SPL's efficacy and efficiency in real-world deployments.

While proxy learning in SNN training is not entirely new, our CIL-SPL method uniquely integrates with hardware, ensuring models are not only theoretically adept but also practically efficient on real-world platforms. Unlike other techniques, CIL-SPL complements existing training methods, thereby bridging the gap between simulation and real-world performance and offering a versatile solution for optimal results.

While CIL-SPL brings forward significant advantages, it is inherently dependent on specific hardware devices or platforms. This means there is no one-size-fits-all solution as different hardware or platforms would necessitate distinct implementations. Additionally, its efficiency is closely tied to the forward inference speed and parallelization capabilities of the chosen hardware or platform. Due to time constraints, our study did not extend to tests on a wider variety of datasets or more diverse hardware platforms. In future endeavors, we aim to delve deeper into these areas and warmly invite fellow researchers and scholars to explore and build upon this foundation.

## Data availability statement

The original contributions presented in the study are available through the public dataset: https://www.garrickorchard.com/datasets/n-mnist.

## Author contributions

YX: Writing – review & editing. YL: Writing – original draft. TL: Writing – original draft. YH: Writing – original draft. WL: Writing – original draft. SS: Writing – review & editing. NQ: Writing – review & editing.

## References

[B1] Abbott L. F. (1999). Lapicque's introduction of the integrate-and-fire model neuron (1907). Brain Res. Bull. 50, 303–304. 10.1016/S0361-9230(99)00161-6 10643408

[B2] Bellec G. Salaj D. Subramoney A. Legenstein R. Maass W. (2018). “Long short-term memory and learning-to-learn in networks of spiking neurons,” in Advances in Neural Information Processing Systems 31.

[B3] Bengio Y. Lee D.-H. Bornschein J. Mesnard T. Lin Z. (2015). Towards biologically plausible deep learning. arXiv preprint arXiv:1502.04156.

[B4] Benjamin B. V. Gao P. McQuinn E. Choudhary S. Chandrasekaran A. R. Bussat J.-M.. (2014). Neurogrid: a mixed-analog-digital multichip system for large-scale neural simulations. Proc. IEEE 102, 699–716. 10.1109/JPROC.2014.2313565

[B5] Davies M. Srinivasa N. Lin T.-H. Chinya G. Cao Y. Choday S. H.. (2018). Loihi: a neuromorphic manycore processor with on-chip learning. IEEE Micro 38, 82–99. 10.1109/MM.2018.112130359

[B6] Delbruck T. (2008). “Frame-free dynamic digital vision,” in Proceedings of International Symposium on Secure-Life Electronics, Advanced Electronics for Quality Life and Society (Citeseer), 21–26.

[B7] Diehl P. U. Cook M. (2015). Unsupervised learning of digit recognition using spike-timing-dependent plasticity. Front. Comput. Neurosci. 9, 99. 10.3389/fncom.2015.00099 26941637 PMC4522567

[B8] Diehl P. U. Neil D. Binas J. Cook M. Liu S.-C. Pfeiffer M. (2015). “Fast-classifying, high-accuracy spiking deep networks through weight and threshold balancing,” in 2015 International Joint Conference on Neural Networks (IJCNN) (IEEE), 1–8. 10.1109/IJCNN.2015.7280696

[B9] Fang W. Yu Z. Chen Y. Masquelier T. Huang T. Tian Y. (2021). “Incorporating learnable membrane time constant to enhance learning of spiking neural networks,” in Proceedings of the IEEE/CVF International Conference on Computer Vision 2661–2671. 10.1109/ICCV48922.2021.00266

[B10] Gallego G. Delbrück T. Orchard G. Bartolozzi C. Taba B. Censi A.. (2020). Event-based vision: a survey. IEEE Trans. Patt. Anal. Mach. Intell. 44, 154–180. 10.1109/TPAMI.2020.3008413 32750812

[B11] Gerstner W. Kistler W. M. (2002). Spiking Neuron Models: Single Neurons, Populations, Plasticity. Cambridge: Cambridge University Press. 10.1017/CBO9780511815706

[B12] Izhikevich E. M. (2003). Simple model of spiking neurons. IEEE Trans. Neural Netw. 14, 1569–1572. 10.1109/TNN.2003.820440 18244602

[B13] Kheradpisheh S. R. Mirsadeghi M. Masquelier T. (2022). Spiking neural networks trained via proxy. IEEE Access 10, 70769–70778. 10.1109/ACCESS.2022.3187033

[B14] LeCun Y. Bottou L. Bengio Y. Haffner P. (1998). Gradient-based learning applied to document recognition. Proc. IEEE 86, 2278–2324. 10.1109/5.726791

[B15] Lee J. H. Delbruck T. Pfeiffer M. (2016). Training deep spiking neural networks using backpropagation. Front. Neurosci. 10, 508. 10.3389/fnins.2016.00508 27877107 PMC5099523

[B16] Liao W. Zhang X. Yu L. Lin S. Yang W. Qiao N. (2022). “Synthetic aperture imaging with events and frames,” in Proceedings of the IEEE/CVF Conference on Computer Vision and Pattern Recognition 17735–17744. 10.1109/CVPR52688.2022.01721

[B17] Maass W. (1997). Networks of spiking neurons: the third generation of neural network models. Neur. Netw. 10, 1659–1671. 10.1016/S0893-6080(97)00011-7

[B18] Merolla P. A. Arthur J. V. Alvarez-Icaza R. Cassidy A. S. Sawada J. Akopyan F.. (2014). A million spiking-neuron integrated circuit with a scalable communication network and interface. Science 345, 668–673. 10.1126/science.1254642 25104385

[B19] Neftci E. O. Mostafa H. Zenke F. (2019). Surrogate gradient learning in spiking neural networks: bringing the power of gradient-based optimization to spiking neural networks. IEEE Signal Proc. Magaz. 36, 51–63. 10.1109/MSP.2019.2931595

[B20] Orchard G. Jayawant A. Cohen G. K. Thakor N. (2015). Converting static image datasets to spiking neuromorphic datasets using saccades. Front. Neurosci. 9, 437. 10.3389/fnins.2015.00437 26635513 PMC4644806

[B21] Pedroni B. U. Sheik S. Joshi S. Detorakis G. Paul S. Augustine C.. (2016). “Forward table-based presynaptic event-triggered spike-timing-dependent plasticity,” in 2016 IEEE Biomedical Circuits and Systems Conference (BioCAS) (IEEE), 580–583. 10.1109/BioCAS.2016.7833861

[B22] Pfeiffer M. Pfeil T. (2018). Deep learning with spiking neurons: opportunities and challenges. Front. Neurosci. 12, 774. 10.3389/fnins.2018.00774 30410432 PMC6209684

[B23] Ponulak F. Kasinski A. (2011). Introduction to spiking neural networks: information processing, learning and applications. Acta Neurobiol. Exper. 71, 409–433. 22237491 10.55782/ane-2011-1862

[B24] Rastegari M. Ordonez V. Redmon J. Farhadi A. (2016). “Xnor-net: imagenet classification using binary convolutional neural networks,” in European Conference on Computer Vision (Springer), 525–542. 10.1007/978-3-319-46493-0_32

[B25] Rathi N. Srinivasan G. Panda P. Roy K. (2020). Enabling deep spiking neural networks with hybrid conversion and spike timing dependent backpropagation. arXiv preprint arXiv:2005.01807.

[B26] Romero A. Ballas N. Kahou S. E. Chassang A. Gatta C. Bengio Y. (2014). Fitnets: hints for thin deep nets. arXiv preprint arXiv:1412.6550.

[B27] Rueckauer B. Lungu I.-A. Hu Y. Pfeiffer M. Liu S.-C. (2017). Conversion of continuous-valued deep networks to efficient event-driven networks for image classification. Front. Neurosci. 11, 682. 10.3389/fnins.2017.00682 29375284 PMC5770641

[B28] Rumelhart D. E. Hinton G. E. Williams R. J. (1986). Learning representations by back-propagating errors. Nature 323, 533–536. 10.1038/323533a0

[B29] Severa W. Vineyard C. M. Dellana R. Verzi S. J. Aimone J. B. (2018). Whetstone: a method for training deep artificial neural networks for binary communication. arXiv preprint arXiv:1810.11521. 10.1038/s42256-018-0015-y

[B30] Shrestha S. B. Orchard G. (2018). “Slayer: spike layer error reassignment in time,” in Advances in Neural Information Processing Systems 31.

[B31] Wei W. Zhang M. Qu H. Belatreche A. Zhang J. Chen H. (2023). “Temporal-coded spiking neural networks with dynamic firing threshold: Learning with event-driven backpropagation,” in Proceedings of the IEEE/CVF International Conference on Computer Vision 10552–10562.

[B32] Williams R. J. Zipser D. (1989). Experimental analysis of the real-time recurrent learning algorithm. Connect. Sci. 1, 87–111. 10.1080/09540098908915631

[B33] Wu J. Chua Y. Zhang M. Li G. Li H. Tan K. C. (2021a). A tandem learning rule for effective training and rapid inference of deep spiking neural networks. IEEE Trans. Neur. Netw. Learn. Syst. 34, 446–460. 10.1109/TNNLS.2021.3095724 34288879

[B34] Wu J. Xu C. Han X. Zhou D. Zhang M. Li H.. (2021b). Progressive tandem learning for pattern recognition with deep spiking neural networks. IEEE Trans. Pattn. Anal. Mach. Intell. 44, 7824–7840. 10.1109/TPAMI.2021.3114196 34546918

[B35] Xing Y. Di Caterina G. Soraghan J. (2020). A new spiking convolutional recurrent neural network (SCRNN) with applications to event-based hand gesture recognition. Front. Neurosci. 14, 590164. 10.3389/fnins.2020.590164 33324153 PMC7722478

[B36] Zhang M. Qu H. Belatreche A. Chen Y. Yi Z. (2018). A highly effective and robust membrane potential-driven supervised learning method for spiking neurons. IEEE Trans. Neur. Netw. Learn. Syst. 30, 123–137. 10.1109/TNNLS.2018.2833077 29993588

[B37] Zhang M. Wang J. Wu J. Belatreche A. Amornpaisannon B. Zhang Z.. (2021). Rectified linear postsynaptic potential function for backpropagation in deep spiking neural networks. IEEE Trans. Neur. Netw. Learn. Syst. 33, 1947–1958. 10.1109/TNNLS.2021.3110991 34534091

